# miR-19b attenuates H_2_O_2_-induced apoptosis in rat H9C2 cardiomyocytes *via* targeting PTEN

**DOI:** 10.18632/oncotarget.7678

**Published:** 2016-02-24

**Authors:** Jiahong Xu, Yu Tang, Yihua Bei, Shengguang Ding, Lin Che, Jianhua Yao, Hongbao Wang, Dongchao Lv, Junjie Xiao

**Affiliations:** ^1^ Department of Cardiology, Tongji Hospital, Tongji University School of Medicine, Shanghai, China; ^2^ Regeneration and Ageing Laboratory, Experimental Center of Life Sciences, School of Life Science, Shanghai University, Shanghai, China; ^3^ Department of Thoracic and Cardiovascular Surgery, The Second Affiliated Hospital of NanTong University, Nantong, China; ^4^ Department of Cardiology, Shanghai Yangpu District Hospital, Tongji University School of Medicine, Shanghai, China

**Keywords:** ischemia reperfusion injury, cardiomyocytes, apoptosis, microRNA, PTEN, Pathology Section

## Abstract

Myocardial ischemia-reperfusion (I-R) injury lacks effective treatments. The miR-17-92 cluster plays important roles in regulating proliferation, apoptosis, cell cycle and other pivotal processes. However, their roles in myocardial I-R injury are largely unknown. In this study, we found that miR-19b was the only member of the miR-17-92 cluster that was downregulated in infarct area of heart samples from a murine model of I-R injury. Meanwhile, downregulation of miR-19b was also detected in H_2_O_2_-treated H9C2 cells *in vitro* mimicking oxidative stress occurring during myocardial I-R injury. Using flow cytometry and Western blot analysis, we found that overexpression of miR-19b decreased H_2_O_2_-induced apoptosis and improved cell survival, while downregulation of that had inverse effects. Furthermore, PTEN was negatively regulated by miR-19b at the protein level while silencing PTEN could completely block the aggravated impact of miR-19b inhibitor on H_2_O_2_-induced apoptosis in H9C2 cardiomyocytes, indicating PTEN as a downstream target of miR-19b controlling H_2_O_2_-induced apoptosis. These data indicate that miR-19b overexpression might be a novel therapy for myocardial I-R injury.

## INTRODUCTION

Acute myocardial infarction represents one of the leading causes of morbidity and mortality worldwide [1]. Although timely reperfusion of the myocardium can limit the infarct area and improve long term cardiac function and survival of the patients, reperfusion itself can also lead to myocardial injury [2, 3]. Despite the successful use of thrombolysis and coronary stenting in the treatment of myocardial ischemia, effective interventions for reducing myocardial reperfusion injury is still lacking [4].

MicroRNAs (miRNAs, miRs) are a group of small non-coding RNAs with ∼22 nucleotides in length, and have been identified as posttranscriptional negative regulators for genes mainly through degradation and/or translational inhibition of target mRNAs [5, 6]. Accumulating evidence show that miRNA dysregulation contributes to multiple cardiovascular diseases including ischemia-reperfusion (I-R) injury [7–11]. miR-1, -26, -29, -21, -24, -103, -133, and -210 have been reported to be regulators of I-R injury either in the early or in the late stage after myocardial infarction, though the underlying mechanisms are largely unclarified [12, 13]. The miR-17-92 cluster is among the best-explored miRNA clusters, including six members (miR-19a, -19b, -17, -18a, -20a, -92a). The miR-17-92 cluster plays important roles in regulating proliferation, apoptosis, cell cycle and other pivotal processes [14]. Dysregulated miR-17-92 cluster has been reported in cardiovascular, immune and neurodegenerative diseases [14]. Although the roles of miR-17-92 in cardiac development, cardiomyocyte proliferation, and cardiac ageing have been reported [14–19], it is still unclear whether, and (if so) how, the miR-17-92 cluster is involved in myocardial I-R injury.

Oxidative stress and apoptosis play fundamental roles in myocardial I-R injury [20–24]. Here we showed that miR-19b was the only member of the miR-17-92 cluster that was downregulated in infarct area of heart samples from a murine model of I-R injury. Meanwhile, decreased miR-19b was also detected in H9C2 cardiomyocytes treated with hydrogen peroxide (H_2_O_2_) mimicking myocardial IRI *in vitro*. Overexpression of miR-19b led to decreased apoptosis and improved survival of H_2_O_2_-treated H9C2 cardiomyocytes. Moreover, PTEN was identified as a target gene of miR-19b that was responsible for its anti-apoptosis effects. These data provide evidence that increasing miR-19b might be a novel therapeutic strategy for reducing cellular apoptosis during myocardial IRI.

## RESULTS

### miR-19b is downregulated in the infarct area of myocardial ischemia-reperfusion mice

**Figure 1:**
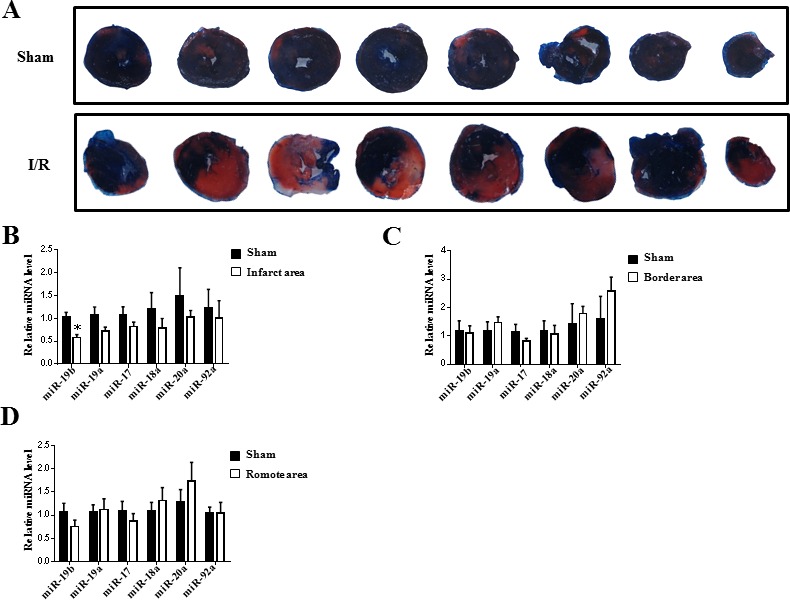
miR-19b is decreased in infarct area of myocardial ischemia-reperfusion mice **A.** TTC staining for heart samples from sham (*n* = 5) and ischemia-reperfusion (I-R) mice (*n* = 5). **B.-D.** qRT-PCR analysis for the miR-17-92 cluster members in heart samples from infarct area, border area and remote area (*n* = 5). *, *P* < 0.05.

Coronary artery ligation for 30 min followed by reperfusion until 24 h was conducted to induce I-R injury in mice. I-R mice displayed marked infarction compared to sham mice, as evidenced by TTC staining (Figure 1A). Using qRT-PCR, miR-19b was found to be the only one in the miR-17-92 cluster that was downregulated in infarct area of heart samples from a murine model of I-R injury (Figure 1B). However, all members of the miR-17-92 cluster remained unchanged in the border area and remote area of heart samples from a murine model of I-R injury (Figure 1C–1D).

### miR-19b is decreased in H_2_O_2_-treated H9C2 cardiomyocytes

**Figure 2:**
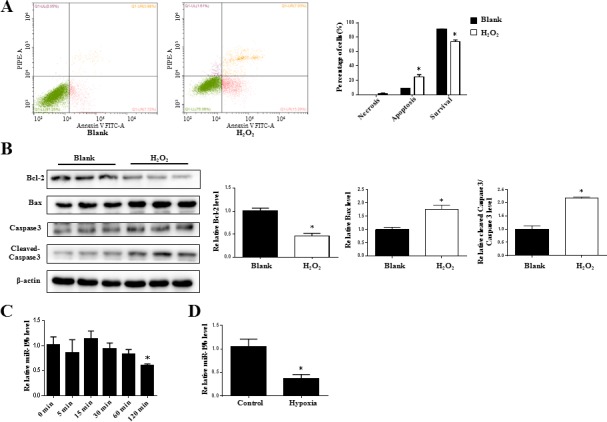
miR-19b is decreased in H_2_O_2_-treated H9C2 cardiomyocytes **A.** Flow cytometry analysis for necrosis, apoptosis, and survival of H9C2 cardiomyocytes treated with H_2_O_2_ (600 μM, 2 h) (*n* = 3). **B.** Immunoblot analysis for Bcl-2, Bax, cleaved-Caspase 3 to Caspase 3 ratio in H_2_O_2_-treated H9C2 cardiomyocytes (600 μM, 2 h) (*n* = 3). **C.** The time-course change of miR-19b as determined by qRT-PCRs in H_2_O_2_-treated H9C2 cardiomyocytes. (*n* = 5) **D.** The downregulation of miR-19b in hypoxia treatment. (*n* = 5) *, *P* < 0.05.

As a common reactive oxygen species, H_2_O_2_ is usually used to mimic I-R injury in *in vitro* experiments. Here we found that H_2_O_2_ treatment for 2 h significantly increased apoptosis in H9C2 cardiomyocytes as analyzed by flow cytometry (Figure 2A) and western blot analysis for Bcl-2, Bax and cleaved-Caspase 3 to Caspase 3 ratio (Figure 2B). The time-course change of miR-19b was determined and miR-19b was found to be downregulated in H_2_O_2_-treated H9C2 cardiomyocytes at 2 h but remained unchanged at 5 min, 15 min, 30 min and 60 min (Figure 2C). Interestingly, hypoxia treatment for 8 h also decreased miR-19b (Figure 2D).

### miR-19b reduces H_2_O_2_-induced apoptosis in H9C2 cardiomyocytes

**Figure 3:**
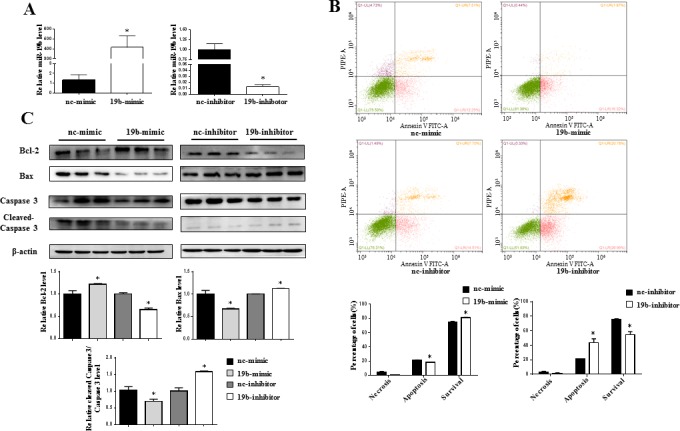
miR-19b reduces H_2_O_2_-induced apoptosis in H9C2 cardiomyocytes **A.** qRT-PCR analysis for miR-19b level in H9C2 cardiomyocytes transfected with miR-19b mimic, inhibitor, or respective negative controls (*n* = 4). **B.** Flow cytometry analysis for necrosis, apoptosis, and survival of H_2_O_2_-treated H9C2 cardiomyocytes with miR-19b overexpression or inhibition (*n* = 4). **C.** Immunoblot analysis for Bcl-2, Bax, cleaved-Caspase 3 to Caspase 3 ratio in H_2_O_2_-treated H9C2 cardiomyocytes with miR-19b overexpression or inhibition (n = 3). NC = negative control. *, *P* < 0.05.

To further examine the functional effect of miR-19b in H_2_O_2_-treated H9C2 cardiomyocytes, transfection of miR-19b mimic, inhibitor, or their negative controls, were conducted. miR-19b mimic was found to be sufficient to increase relative miR-19b level, while miR-19b inhibitor had inverse effect, confirming that miR-19b mimic and inhibitor took effects in H9C2 cardiomyocytes (Figure 3A). Flow cytometry showed that miR-19b mimic reduced H_2_O_2_-induced apoptosis in H9C2 cardiomyocytes, while miR-19b inhibitor aggravated that (Figure 3B). Meanwhile, miR-19b overexpression led to increased expression of Bcl-2, decreased expression of Bax, and reduced cleaved-Caspase 3 to Caspase 3 ratio at protein levels, while miR-19b inhibition had inverse effects (Figure 3C). These data indicate a protective effect of miR-19b against H_2_O_2_-induced apoptosis in H9C2 cardiomyocytes.

### PTEN is a downstream target of miR-19b controlling H_2_O_2_-induced apoptosis in H9C2 cardiomyocytes

**Figure 4:**
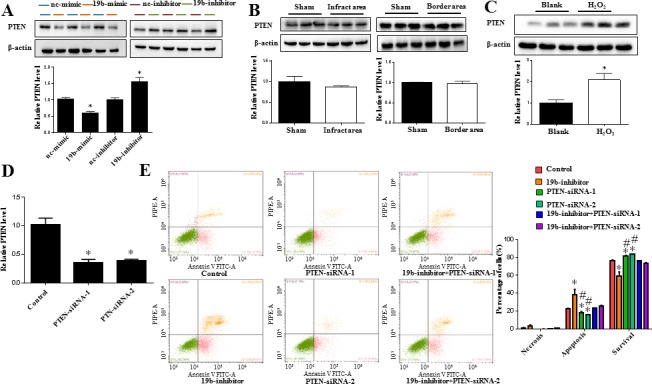
PTEN is a target gene of miR-19b controlling H_2_O_2_-induced apoptosis in H9C2 cardiomyocytes **A.** Immunoblot analysis for PTEN protein level in H9C2 cardiomyocytes transfected with miR-19b mimic, inhibitor, or respective negative controls (*n* = 3). **B.** Immunoblot analysis indicated that PTEN was not changed in infarct area and border area myocardium (*n* = 3). **C.** Immunoblot analysis showed that PTEN was upregulated in H_2_O_2_-treated H9C2 cardiomyocytes (*n* = 3). **D.** qRT-PCRs confirmed that siRNAs for PTEN successfully decreased PTEN at least at the mRNA level (*n* = 4). **E.** Flow cytometry analysis for necrosis, apoptosis, and survival of H_2_O_2_-treated H9C2 cardiomyocytes with miR-19b inhibition and/or PTEN silence (*n* = 4). NC = negative control. *, *P* < 0.05 *vs.* control; #, *P* < 0.05 *vs.* miR-19b inhibitor.

**Figure 5:**
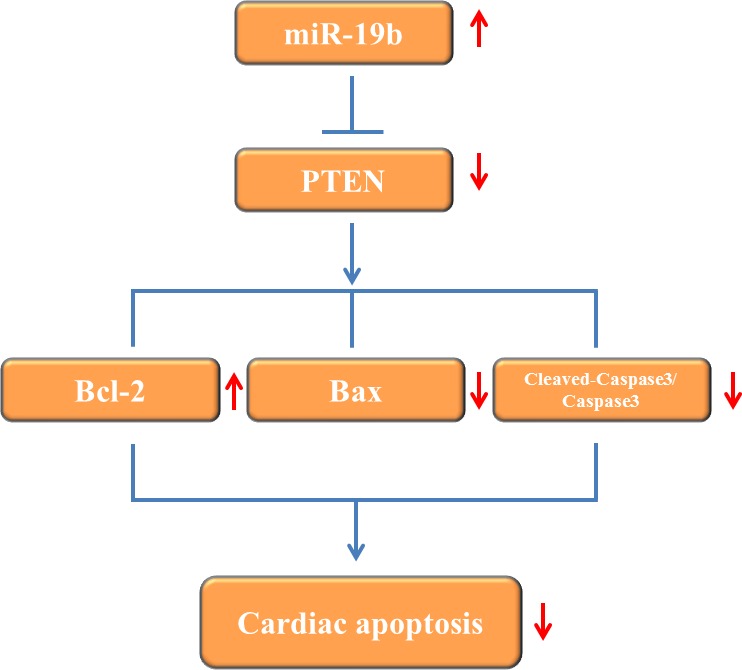
Proposed mechanisms by which miR-19b protects apoptosis induced by H_2_O_2_ in H9C2 cardiomyocytes

How miR-19b modulates H_2_O_2_-induced apoptosis in H9C2 cardiomyocytes was examined. PTEN is a well-known target gene of miR-19b [25–27]. In the current study, immunoblot analysis showed that PTEN was inversely regulated by miR-19b in H9C2 cells (Figure 4A). In addition, although PTEN was not changed in infarct area and border area myocardium (Figure 4B), it was significantly upregulated in H_2_O_2_-treated H9C2 cardiomyocytes (Figure 4C). Moreover, silencing PTEN alone led to reduced apoptosis and improved cell survival in H_2_O_2_-treated H9C2 cardiomyocytes, while co-transfection of PTEN-siRNA and miR-19b inhibitor could totally abolish the aggravated effect of miR-19b inhibitor on cell apoptosis in H9C2 cardiomyocytes treated with H_2_O_2_ (Figure 4D–4E). These data suggest that PTEN is responsbile for the effects of miR-19b in H2O2-induced apoptosis in H9C2 cardiomyocytes (Figure 5).

## DISCUSSION

Myocardial I-R injury is a detrimental process, usually leading to increased infarct size, impaired cardiac function, and even fibrotic hypertrophy and cardiac remodeling over time [28, 29]. However, effective interventions for myocardial I-R injury are still lacking [28, 29]. In the present study, we found that miR-19b was the only one among the miR-17-92 cluster that was decreased in infarct area of heart samples from a murine model of I-R injury. Meanwhile, miR-19b was also downregulated in H_2_O_2_-treated H9C2 cardiomyocytes, and miR-19b overexpression was sufficient to reduce H_2_O_2_-induced cardiomyocyte apoptosis. Furthermore, PTEN was identified as a downstream target of miR-19b controlling apoptosis in H_2_O_2_-treated cardiomyocytes. Therefore, our data suggest that miR-19b might be a novel therapeutic target for reducing early cellular apoptosis during myocardial I-R injury.

Many pathophysiological mechanisms are supposed to be responsible for myocardial I-R injury, including oxidative stress, cell death, calcium overload, and pressure/mechanical stress [3, 20, 21]. Actually, it has been widely accepted that oxidative stress and cell apoptosis are key contributor factors for I-R injury [22, 30]. Previously, multiple miRNAs have been reported to be involved in acute myocardial infarction and myocardial I-R injury [12, 31]. Nevertheless, the roles of miRNAs in oxidative stress-associated cardiomyocyte apoptosis are far from elucidated. Here, we found that miR-19b was markedly downregulated in infarct area heart samples from a murine model of I-R injury. As a key functional member of miR-17-92 cluster, miR-19b has emerging roles implicated in cardiac physiology and pathophysiology changes [15–19]. To the best of our knowledge, we firstly reported a downregulation of miR-19b in myocardial I-R injury, which promoted us to further determine the underlying mechanisms.

Knowing that during the early stage (first 24 h after ischemia) of myocardial I-R injury, production of reactive oxidative species triggers and further enhances cardiomyocyte apoptosis, we continued to determine the functional roles of miR-19b on H_2_O_2_-induced apoptosis in rat H9C2 cardiomyocytes. As a key by-product of I-R injury, H_2_O_2_ is commonly used to induce oxidative stress-associated apoptosis in H9C2 cardiomyocytes [32, 33]. Several lines of evidence indicates that miRNAs regulate apoptosis in H_2_O_2_-treated H9C2 cardiomyocytes. It has previously been reported that upregulating miR-21 or downregulating miR-181a reduces H_2_O_2_-induced apoptosis in H9C2 cardiomyocytes, and that overexpressing miR-210 contributes to the protective effect of insulin against apoptosis in H_2_O_2_-treated H9C2 cardiomyocytes [34–36]. Besides the reduction of miR-19b expression in infarct area of heart samples from I-R mice, we also found that miR-19b was downregulated in H_2_O_2_-treated H9C2 cardiomyocytes. Furthermore, our data demonstrated that miR-19b overexpression reduced H_2_O_2_-induced apoptosis and improved cell survival in H9C2 cardiomyocytes, accompanied with increased expression of Bcl-2 and decreased expression of Bax and cleaved-Caspase 3/Caspase 3 ratio, indicating that miR-19b overexpression might provide protective effects against oxidative stress-related cellular apoptosis.

PTEN is a well-known target gene of miR-19b, which mainly regulates cancer cell growth and proliferation [37–40]. Meanwhile, PTEN overexpression has been reported to increase cellular apoptosis [41–45]. Based on that, we proceeded to investigate whether PTEN could be a downstream effector of miR-19b mediating its effect in H_2_O_2_-induced apoptosis. As expected, PTEN was negatively regulated by miR-19b at the protein level in H9C2 cardiomyocytes. Importantly, silencing PTEN could abolish the aggravated apoptotic effect of miR-19b inhibitor in H_2_O_2_-treated H9C2 cardiomyocytes. These data suggest that PTEN is a downstream effector of miR-19b controlling cardiomyocyte apoptosis-induced by H_2_O_2_.

In conclusion, miR-19b overexpression is able to attenuate apoptosis in H_2_O_2_-treated H9C2 cardiomyocytes. PTEN is a target gene of miR-19b, responsible for the anti-apoptosis effect of miR-19b. Therefore, miR-19b overexpression might be a novel therapy for myocardial I-R injury.

## MATERIALS AND METHODS

### Animal model

Eight-week-old male C57BL/6 mice were purchased from Shanghai Laboratory Animal Center (SLAC, Shanghai, China) and housed in autoclaved ventilated cages with sterile food and water *ad libitum*. All animal experimentations were conducted under the established guidelines on the use and care of laboratory animals for biomedical research published by National Institutes of Health (No. 85-23, revised 1996). To induce myocardial I-R injury, mice were subjected to 30 min of coronary artery ligation followed by reperfusion until 24 h. In brief, after anesthetized with ketamine and sevoflurane, the ligation of left coronary artery was conducted about 2 mm under the left auricle using 7-0 silk sutures. After 30 min of ischemia, reperfusion was carried out until 24 h when mice were sacrificed. The sham mice underwent left thoracotomy only. Myocardial infarct size was analyzed using triphenyltetrazolium chloride (TTC) staining. Heart samples were collected according to samples from infarct area, border area and remote area. All samples were harvested and snap frozen in liquid nitrogen until RNA extraction.

### Cell culture and H_2_O_2_ treatment

Rat cardiomyocyte H9C2 cell line was purchased from the Cell Bank of Chinese Academy of Science (Shanghai, China) and cultured in Dulbecco's modified eagle's medium (DMEM, Corning, USA) supplemented with 10% fetal bovine serum (Biolnd, Israel) and 1% streptomycin/penicillin in a humidified atmosphere containing 5% CO_2_ at 37°C. H9C2 cardiomyocytes were treated with 600 μM H_2_O_2_ (Sigma, USA) for 2 h to induce cell apoptosis mimicking I-R injury *in vivo*. In addition, to determine the time-course of miR-19b changes, H9C2 cardiomyocytes were also treated with 600 μM H_2_O_2_ from 0 min to 120 min. Moreover, hypoxia treatment (0% O_2_) was also conducted in H9C2 cardiomyocytes for 8 h.

### Cell transfection

The miR-19b mimic, inhibitor, and their negative controls, as well as PTEN-siRNAs, were all purchased from RiboBio (Guangzhou, China). Cells were transfected after 8 h of starvation. To overexpress or inhibit miR-19b expression, miR-19b mimic (50 nM), inhibitor (100 nM), or respective negative controls was transfected to H9C2 cardiomyocytes for 48 h using lipofectamine 2000 (Invitrogen, USA). To silence PTEN expression, PTEN-siRNAs (100 nM) were transfected to H9C2 cardiomyocytes for 48 h using lipofectamine 2000 in accordance with the manufacturer's instruction.

### Flow cytometry detection of cell apoptosis and necrosis

After transfected with miR-19b mimic, inhibitor, or respective controls for 48 h, cell apoptosis and necrosis were analyzed using Annexin V-FITC and propidium iodide (PI) kit (Dojindo, Japan) according to the manufacturer's instruction, followed by flow cytometry analysis (Beckman Coulter, USA).

### Quantitative reverse transcription-polymerase chain reaction (qRT-PCR)

Total RNAs were isolated using Trizol Reagent (Invitrogen, USA) and reverse transcribed into cDNA using iScript^TM^ cDNA synthesis kit (Bio-Rad, USA). For detection the mRNA level of PTEN, the RT product was subjected to 40 cycles of quantitative PCR with Takara SYBR Premix Ex Taq^TM^ (Tli RNaseH Plus, Japan) in CFX96^TM^ Real-Time PCR Detection System (Bio-Rad, USA). 18s was used as an internal reference. The primer sequences (forward and reverse) are as follows: PTEN, CAATGTTCAGTGGCGAACTT and GGCAATGGCTGAGGGAACT; 18s, ATTC GAACGTCTGCCCTATCAA and CGGGAGTGGGTAATTTGCG. For quantitative miRNA analysis, Bulge-Loop™ miRNA qPCR Primer Set (RiboBio, China) was used to detect miRNA expression levels with Takara SYBR Premix Ex Taq^TM^ (Tli RNaseH Plus, Japan) by qRT-PCR in CFX96^TM^ Real-Time PCR Detection System. 5s was used for normalization of miRNA expression levels. The relative expression levels for each mRNA and miRNAs were calculated by the 2^−ΔΔCT^method.

### Western blot analysis

Samples were lysed in RIPA buffer (KeyGen, China) containing 1% phenylmethanesulfonyl fluoride (PMSF). Total proteins were quantified with the BCA protein assay kit (KeyGen, China). Lysates equivalent to 30 μg of protein were subjected to 10% SDS-PAGE gels, transferred onto PVDF membranes, and probed with primary antibodies anti-PTEN (1:1000; Epitomics, ab154812), anti-Bcl-2 (1:1000; Abclonal, A0208), anti-Bax (1:1000; Abclonal, A2211), and anti-Caspase-3 (1:1000; Abclonal, A2156) overnight at 4°C. β-actin (1:10000; Abclonal, AC004) was used as a loading control. After incubation with appropriate secondary antibodies for 2 h at room temperature, ECL System (Bio-Rad, USA) was used to visualize the protein bands with ChemiDoc^TM^ XRS Plus luminescent image analyzer (Bio-Rad, USA).

### Statistical analysis

Results were presented as mean±SEM. All data were analyzed with an independent-samples T-test or one-way ANOVA test followed by Bonferroni's tests using SPSS (version 19). *P* < 0.05 was regarded as statistical significance.
